# A multigroup structural equation modeling analysis of students’ perception, motivation, and performance in computational thinking

**DOI:** 10.3389/fpsyg.2022.989066

**Published:** 2022-09-07

**Authors:** Jiachu Ye, Xiaoyan Lai, Gary Ka Wai Wong

**Affiliations:** Faculty of Education, The University of Hong Kong, Hong Kong, Hong Kong SAR, China

**Keywords:** computational thinking, perception, motivation, gender difference, structural equation model

## Abstract

Students’ perceptions of learning are important predictors of their learning motivation and academic performance. Examining perceptions of learning has meaningful implications for instruction practices, while it has been largely neglected in the research of computational thinking (CT). To contribute to the development of CT education, we explored the influence of students’ perceptions on their motivation and performance in CT acquisition and examined the gender difference in the structural model using a multigroup structural equation modeling (SEM) analysis. Two hundred and eighty-five students from a Chinese urban high school were recruited for the study. The analysis revealed that students’ perceptions of CT positively influenced their CT performance and learning motivation, and some motivational constructs, namely self-efficacy and learning goal orientation (LGO), also positively influenced their CT performance. Furthermore, in the male student group, perceptions of CT exhibited significant correlations with both self-efficacy and LGO. However, no significant correlation was found in the female student group. Implications for research and teaching practice in CT education are presented herein.

## Introduction

Computational thinking (CT) is a fundamental problem-solving skill in the twenty first century that can be applied to various educational disciplines and daily life (Vallance and Towndrow, 2020; Kafai and Proctor, 2022). Fostering students’ CT development is a significant goal in the field of computer science (CS) education (Barr and Stephenson, 2011; Chen et al., 2017). Proposed by Papert (1980) and popularized by Wing (2006), CT has attracted increasing research attention, with numerous studies focusing on its conceptualization, interventional approaches, and methods of assessment (Lye and Koh, 2014; Shute et al., 2017; Tang et al., 2020).

Besides the development of CT pedagogies, researchers have started to examine cognitive and affective factors in the process of CT acquisition (Tang et al., 2020). The perception of CT is an essential factor yielding meaningful insights into CT teaching and learning practices (Kong and Wang, 2019). Research has shown that perceptions of learning and motivation are highly correlated in various learning contexts and that these measures are also significant predictors of learning performance (Mayer, 2009). Understanding the effects of perceptions of learning and motivation to learn will provide insights into more effective learning strategies for students and educators as well as benefit the implementation of CT education programs. Currently, some empirical studies have investigated teachers’ perceptions of CT and the influence on their teaching practices (Ling et al., 2018; Sands et al., 2018; Kong and Wang, 2019), but students’ perceptions of CT have been visited less frequently (Polat et al., 2021), and how students’ perceptions generate impacts on their motivation and performance in CT remains unknown. Therefore, in this study, we attempted to address the aforementioned research gap by examining the influence of high school students’ perceptions of CT on their motivation and performance in CT acquisition.

## Related works

### Perceptions of computational thinking

According to Angell (1904), perception is the “consciousness of particular material things present to sense.” Similarly, Lindsay and Norman (1977) defined perception as the process by which people “interpret and organize sensation to produce a meaningful experience of the world” based on their information and understanding. Perception relates to how people perceive and interpret any object. Understanding individuals’ perception is crucial to educational practices, as research has shown that perception can serve as a predictor of learners’ motivation to learn and expectation of learning (Martens et al., 2007) and that it can help deepen the understanding of their learning attitude and knowledge (Maio and Haddock, 2009).

In the field of CT and programming education, several attempts have been made to explore the idea of perception and its relationships with other cognitive and affective factors. To measure teachers’ perception of programming, Kong and Wang (2019, 2020) created a questionnaire scale called Perception of Programming Education, measuring from three dimensions: programming understanding, programming expectation, and programming support. Sands et al. (2018) developed a survey on teachers’ perceptions of CT to explore the activities that teachers perceive to be involved in CT. The CT Scales (Korkmaz et al., 2017) is a validated questionnaire that is occasionally used to measure students’ CT perceptions. Nonetheless, not many studies have conceptualized perception using detailed constructs, and within the limited number of studies on students’ perceptions of CT and programming, most of them examined the concept of perception using binary categories (e.g., “positive and negative” and “like and dislike”) via self-designed questionnaires or interview questions (Zainal et al., 2012; Adler and Kim, 2018; Wong and Cheung, 2020).

Research has shown that a positive perception of learning is correlated with motivation as well as learning performance in various contexts. Miyazoe and Anderson (2010) analyzed students’ perceptions of learning English through questionnaires and noticed that students with higher levels of perceived usefulness of English language learning performed better in English writing tests. Fabian et al. (2018) conducted a correlation test, and the results showed that students’ positive perceptions of mathematics learning activities also lead to higher achievement in mathematics. In programming education, research has revealed the significant influence of perceived programming skills on learning motivation, with pre-course perceptions having no overall influence on students’ performance (Zainal et al., 2012). Few empirical studies have explored the relationships among perceptions, motivation, and performance in CT.

Instead of using a simple binary definition of perception, in this current study, we examine the perceptions of CT from three sub-dimensions: perceived impact, perceived interest, and perceived utility, which are significant factors of perception constructs (Kong and Wang, 2019). The perceived impact of CT refers to the degree to which accomplishing a task is perceived to “make a difference in the scheme of things” (Frymier et al., 1996). Perceived interest in CT refers to the degree to which students show positive feelings toward, greater concentration on, and an enduring predisposition toward CT learning activities (Kong et al., 2018). Perceived utility of CT refers to the degree to which students perceive CT fitting into their future academic and career plans (Wigfield and Cambria, 2010).

### Motivation

Taking various aspects of learning motivation into consideration facilitates the study of complex learning environments (Mayer, 2009). In this study, we considered the concept of motivation from the following four motivational constructs: self-efficacy, learning goal orientation (LGO), performance goal orientation (PGO), and learning value. All of these constructs have been suggested as potential determinants of academic success (Nolen and Haladyna, 1989; Tuan et al., 2005; Schunk and Zimmerman, 2012).

Self-efficacy refers to people’s belief in their capacity to “execute behaviors necessary to produce specific performance attainments” (Bandura, 1986). Considerable empirical research has indicated the significant positive influence of self-efficacy on students’ learning performance in the contexts of mathematics (Schöber et al., 2018), science (Aurah, 2017; Uçar and Sungur, 2017), and language learning (Namaziandost and Çakmak, 2020). However, the findings on the relationship between self-efficacy and learning performance in the context of CT are inconsistent. In a quasi-experimental study, CT education through digital programming story design has been shown to be effective in increasing both learners’ self-efficacy and learning achievement (Durak, 2018; Durak et al., 2019). The effectiveness of promoting CT through robotics activities has also been validated based on a learning intervention study (Jaipal-Jamani and Angeli, 2017). However, empirical evidence from an experimental study indicated a low correlation between CT and self-efficacy for elementary school students (Wei et al., 2021).

Goal orientation is described as one’s goal preferences in learning or achievement contexts (Payne et al., 2007), and it is usually distinguished into LGO and PGO. LGO refers to students’ preference for self-development through the acquisition of new skills, the mastery of new situations, and the improvement of their competencies, whereas PGO refers to students’ preference for peer competition and teacher attention during the learning process (VandeWalle and Cummings, 1997). Goal orientation is not a bipolar construct, with research suggesting that students can simultaneously have high levels of LGO and PGO (Button et al., 1996). Numerous studies have shown that LGO has positive effects on learning performance, whereas studies on PGO have revealed mixed effects. Through the comparison of pre- and post-test results, Gerhardt and Brown (2006) showed that higher levels of LGO result in higher levels of self-efficacy and better performance, whereas higher levels of PGO lead to a decrease in self-efficacy and performance. From the analysis of students with different levels of academic performance, Hsieh et al. (2007) also suggested that LGO is significantly positively related to students’ academic standing, but PGO shows no such relationship.

In the context of programming education, empirical research has proven that higher levels of LGO result in better programming performance (Bergin et al., 2005). Research in the context of a programming massive open online course (Polso et al., 2020) indicated that students with higher levels of combined LGO and PGO perform better than others, albeit slightly. However, Yukselturk and Bulut (2005) found inconsistent results: in their first course, LGO was positively correlated with programming achievement, but PGO was negatively correlated with programming achievement; in the second course, both LGO and PGO were positively correlated with programming achievement. In the context of CT, Gong et al. (2020) showed that overall learning motivation significantly influences CT skills. However, they did not more closely examine goal orientation and CT skills.

The value of developing CT skills for students includes but is not limited to acquiring CT competencies, experiencing computational problem-solving activities, stimulating their thinking, and identifying the connections between CT and daily life (Wing, 2011; Grover and Pea, 2013). Learning value, also interchangeably referred to as task value, is a key motivational construct, and it has shown a significant correlation with expectancy and knowledge learning (Eccles and Wigfield, 1995; Wigfield et al., 2012).

Bong (2001) found learning value to be a predictor of students’ academic performance, whereas Neuville et al. (2007) showed that learning value could only influence students’ future course enrolment intention and was not correlated with academic performance. In the context of CS education, empirical studies have indicated that students with high levels of learning value perform better in programming (Bergin et al., 2005; Román-González et al., 2018). However, some studies have presented contradictory results, showing that learning value has no significant relationship with programming performance (Watson et al., 2014). More recently, Guggemos (2021) examined various predictors of CT for high school students and found that learning value can predict students’ level but not their development of CT skills.

Inconsistent findings of motivational constructs in the CT context call for further investigation. Therefore, we conducted this study from the alternative perspective of students’ perception of CT skills, and we examined the influence of this perception on students’ motivational constructs and performance in the CT acquisition process.

### Gender difference

The empirical research on CT education has revealed some gender differences in students’ affective and motivational development. In a game-based CT instruction workshop, Horn et al. (2016) discovered that, for middle school students, males were more interested in topics like modern technology and adventure stories, but females showed more interest in language literature-related topics. Angeli and Valanides (2020) found that males benefited more from kinesthetic and manipulative learning activities while females were more engaged in collaborative writings. In terms of motivation to learn, Sullivan et al. (2015) noticed that through an intervention in a programming club, male students demonstrated significantly higher levels of self-efficacy than female students in CS. In addition, they found that after the intervention, males expressed stronger aspirations to pursue a bachelor’s degree in CS, but females did not report the same intention. More recently, Wei et al.’s (2021) study echoed previous empirical evidence that male students’ self-efficacy in learning CT could be improved significantly through collaborative programming activities, while female students showed no significant effect.

Furthermore, Research has indicated inconsistent results in the development of CT across gender groups. Some studies reported that male and female students had no significant differences in cognitive learning outcomes in CT (Sullivan and Bers, 2013; Buffum et al., 2016; Chou, 2020). However, conflicting results also existed. Dagienë et al. (2015) reported that male students performed better in tasks requiring spatial thinking skills while female students performed better in tasks with clear instructions in CT assessments. Similarly, Kim et al. (2021) revealed that in a CT literacy test, male students scored higher in programming skills, but female students performed better in abstraction skills. More research on the development of CT across genders is required.

### Research questions

Based on the foregoing discussion, we explore the following research questions: (a) What are the effects of high school students’ perceptions of CT on different CT learning motivational factors (i.e., self-efficacy, learning values, LGO, and PGO) and on CT learning performance? (b) What are the effects of different motivational factors on high school students’ CT learning performance? (c) Do the effects stated above differ by gender?

### Hypothesized structural model

**FIGURE 1:**
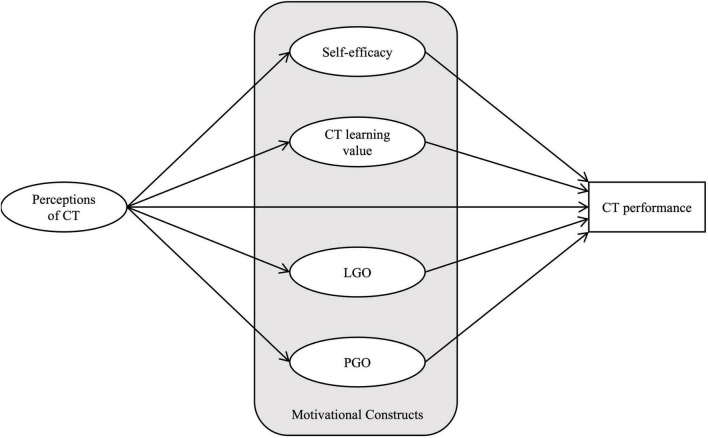
Hypothesized structural model.

Based on review studies, we hypothesize the causal model presented in Figure 1 to explore the direct effects of the perceptions of CT on motivational factors (i.e., self-efficacy, LGO, PGO, and learning value) and on students’ learning performance in CT. We also examined the potential effect of motivational factors on students’ learning performance in CT. We further examined whether the structural model differs across different gender groups.

We hypothesize that (a) the perceptions of CT positively influence all four motivational factors (i.e., self-efficacy, learning values, LGO, and PGO) and students’ CT performance. We also hypothesize that (b) students’ self-efficacy, LGO, PGO, and learning value positively influence their CT performance. In addition, we hypothesize that (c) there is no significant difference in the structural model coefficients between the male and female student groups.

## Methodology

### Procedure

This study was conducted in an urban high school in China. Through convenience sampling, students from Grades 10 and 11 were invited to voluntarily participate in the study. The student demographic comprised mostly Chinese students (99%) along with students from other races (1%). Of the 285 students recruited, 144 (50.53%) were male, 141 (49.47%) were female, and their average age was 16. The school had implemented a CS curriculum called Information Technology; CT is embedded in the curriculum through learning Python programming. The participating students had taken the programming course once per week for 3 months and had a basic knowledge of Python programming and CT concepts. For the data collection, two questionnaires on perceptions and learning motivation of CT and one performance test of CT were conducted. All questionnaires and test items used in the experiment were in Chinese in consideration of students’ language preferences. Participants were asked to indicate their gender (1 = male, 2 = female) as demographic information at the beginning of the questionnaires.

Kline (2016) proposed that sample sizes over 200 can be considered large for a structural equation modeling (SEM) analysis. Bentler and Chou (1987) also suggested a sample size to estimated parameters (N:p) ratio of 5:1 as acceptable for a structural equation model. The 46 free parameters and 285 participants in this study yielded a ratio of 6.2:1. Therefore, based on the criteria, the sample size in this study was sufficient to address the hypotheses.

### Measures

#### Perceptions of computational thinking

The students’ perceptions of CT were measured using a questionnaire adapted from the programming empowerment questionnaire (Kong et al., 2018) and the Coding Attitude Survey (Mason and Rich, 2020) on a 5-point Likert scale (1 = strongly disagree; 5 = strongly agree). The two questionnaires were designed to assess students’ perceived meaningfulness, impact, and interest in computer programming (Kong et al., 2018; Mason and Rich, 2020). Programming is closely associated with CT and is an important interventional approach for CT development (Shute et al., 2017). In addition, the target participants of two questionnaires were senior primary school students, which ensured secondary school students could easily understand the question items without confusion. The participants of the current study are familiar with programming and CT due to their prior learning experience. Therefore, the questionnaires are effective tools for measuring students’ perceptions of CT. The reported reliability values (Cronbach’s alpha) for each construct in the original questionnaires were above 0.70 (Kong et al., 2018; Mason and Rich, 2020), which suggested good reliability. In this current study, the scale consisted of 12 items, with four items measuring the students’ perceived impact of CT (e.g., “We can use CT to solve many problems in the world”), four items measuring the students’ perceived interest in CT (e.g., “I am curious about the content of CT”), and four items measuring the students’ perceived utility of CT (e.g., “I can use CT in other school subjects”).

We applied the internal consistency parceling method for the multidimensional item set (Kishton and Widaman, 1994), in which the mean of the four items in one construct was used to indicate each parcel, and three observed variables were obtained for students’ perceptions of CT. The data parceling method was used considering its psychometric and estimation advantages. Previous research in favor of parcels has highlighted the psychometric merits of data parceling, such as higher reliability, higher communality, and a lower likelihood of distributional violation (Bagozzi and Heatherton, 1994; Kishton and Widaman, 1994). Furthermore, data parceling benefits the model estimation because models from parceled data are more parsimonious and produce fewer sampling errors in various sources (Little et al., 2002).

#### Motivation

Four motivational constructs were measured in this study: self-efficacy, LGO, PGO, and CT learning value. The questionnaire was adapted from the students’ Motivation Toward Science Learning Questionnaire (Tuan et al., 2005) and the Motivated Strategies for Learning Questionnaire (Pintrich and de Groot, 1990). The two questionnaires are influential and effective tools for assessing students’ motivation in various subject areas. They have also been administered in a wide range of student populations from primary to higher education levels (Ke, 2014; Guven et al., 2020). In this current study, a 5-point Likert scale (1 = strongly disagree; 5 = strongly agree) was also used, and there were three questions per construct. Sample items included “I am confident about understanding CT concepts” (self-efficacy), “I feel most fulfilled when I am able to solve a difficult CT problem” (LGO), “I participate in the programming course to perform better than other students” (PGO), and “I think that it is important to learn to solve computational problems in developing CT” (learning value).

#### Computational thinking performance

The students’ CT performance was measured using items adapted from the CT Test (Román-González et al., 2017) and self-designed questions on basic CS concepts. CT is regarded as a learning product in the CT Test, which uses multiple-choice questions to evaluate students’ programming knowledge as the evidence of students’ CT proficiency (Tang et al., 2020). The target school levels for the test are primary and secondary schools. The self-designed questions were designed by the Informational Technology course instructors of the participating school. In this current study, the performance test comprised 20 question items in total, of which six self-designed items assessed the students’ understanding of CS concepts, and 14 items assessed the students’ CT skills, which include sequences, loops, conditionals, and functions in a block-based programming environment. The maximum score was 120 points. Cronbach’s alpha for the performance test was 0.88.

### Data analysis

IBM SPSS Amos 27 software (Arbuckle, 2020) was used for the SEM analysis. Maximum likelihood estimation was used in the analysis. A confirmatory factor analysis was conducted for the measurement model for the five latent variables (perceptions of CT, self-efficacy, LGO, PGO, and CT learning value). Cronbach’s alpha, the composite reliability (CR), and the average variance extracted (AVE) were reported. Cronbach’s alpha and CR are commonly used methods to assess the reliability of questionnaires. CR performs better than Cronbach’s alpha in questionnaires with multidimensional scales (McNeish, 2018). AVE is usually used to measure discriminant validity in SEM analysis (Fornell and Larcker, 1981), which helps explain how much of the variations in the item can be explained by the construct. Given that Cronbach’s alpha coefficients require very strict assumptions and perform better for unidimensional scales (McNeish, 2018), we further presented alternative indicators, CR and AVE, for the reliability of scales.

The structural model was then tested, and the Chi-square test results and other fit indices, namely the comparative fit index (CFI), the goodness-of-fit index (GFI), the adjusted GFI (AGFI), and the root mean square error of approximation (RMSEA), were reported. The Chi-square test is a commonly used global fit index in SEM (Kline, 2016). Besides the Chi-square test, model fit indices mentioned above are also indicators for model evaluation. Specifically, CFI compares the fit of a hypothesized model with the fit of a baseline model; GFI and AGFI represent the proportion of variance accounted for by estimated covariance; RMSEA indicates how far the hypothesized model is from the perfect model (Kline, 2016). Noted that researchers have revealed that Chi-square is sensitive to sample size, and “a Chi-square will almost always be significant (indicating a poor fit) even with only modest sample sizes” (Iacobucci, 2010). It is suggested that if the statistic adjusted by its degrees of freedom was less than 3 (i.e., χ^2^/*df* ≤ 3), the model indicates a reasonable fit (Kline, 2016). In the path analysis of the structural model, we reported the path coefficients (i.e., β weights), which indicated the direct effect of one variable on the other variable in the model (Kline, 2016).

**FIGURE 2:**
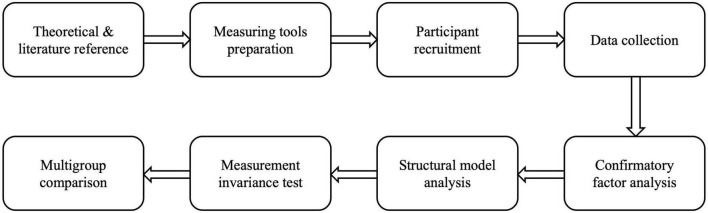
Procedure flowchart of the research.

Next, measurement invariance and structural invariance analyses were conducted for the multigroup comparison. Different levels of measurement invariance were reported as a prerequisite of the multigroup comparison, including the configural invariance (i.e., free estimation of all parameters across groups without constraint), the metric invariance (i.e., free estimation of parameters across groups except for factor loadings), and the scalar invariance (i.e., free estimation of parameters across groups except for factor loadings and item intercepts). The Satorra–Bentler scaled Chi-square difference was reported (Satorra and Bentler, 2010), and other criteria [ΔCFI, ΔTLI (Tucker-Lewis Index), and ΔRMSEA] were also included (Little, 1997; Cheung and Rensvold, 2002; Chen, 2007). A structural invariance test was then performed. A procedure flowchart of the research is presented in Figure 2.

## Results

### Descriptive statistics

Table 1 presents the descriptive statistics of the study variables, namely the sample size, mean, standard deviation, skewness, and kurtosis. The skewness values were between −3 and +3, and the kurtosis values were between −10 and +10, suggesting that the data followed the normal distribution (Kline, 2016).

**TABLE 1:** Descriptive statistics.

Variables		Mean	SD	Skewness	Kurtosis
Perceptions	Item1	4.13	0.833	–0.95	1.32
	Item2	4.18	0.812	–1.042	1.749
	Item3	4.23	0.805	–1.164	2.125
Self-efficacy	Item1	2.8	1.142	0.333	–0.688
	Item2	2.79	1.137	0.376	–0.631
	Item3	2.74	1.146	0.421	–0.625
LGO	Item1	2.98	1.112	0.173	–0.595
	Item2	2.98	1.097	0.202	–0.514
	Item3	2.93	1.125	0.235	–0.664
PGO	Item1	4.08	0.751	–0.476	0.127
	Item2	4.09	0.718	–0.313	–0.481
	Item3	4.07	0.718	–0.39	0.172
Learning value	Item1	4.09	0.689	–0.18	–0.669
	Item2	4.02	0.724	–0.204	–0.599
	Item3	4.07	0.701	–0.154	–0.763
CT performance		72.787	12.7515	–0.106	0.208

### Confirmatory factor analysis

By intercorrelating all latent variables (i.e., perceptions of CT, self-efficacy, LGO, PGO, and CT learning value), a confirmatory factor analysis was performed in the overall sample. Although the Chi-square result was significant, the measurement model indices were within the recommended criteria and indicated a good fit to the data (χ^2^ = 140.25, *df* = 80, *p* < 0.001, χ^2^/*df* = 1.753, GFI = 0.938, AGFI = 0.907, CFI = 0.987, RMSEA = 0.051).

Table 2 shows the standardized factor loadings of each item as well as the Cronbach’s alpha, the CR, and the AVE for each construct. Cronbach’s alphas higher than 0.90 and CR values greater than 0.60 suggest that the measurement model has good reliability (Bagozzi and Yi, 1988). Standardized factor loadings greater than 0.70 and AVE values greater than 0.50 suggest that the measurement model has good convergent validity (Bagozzi and Yi, 1988; Hair et al., 2006). Table 3 shows that the measurement model has good discriminant validity, as the square root of the AVE for each construct is greater than its correlation with other constructs (Fornell and Larcker, 1981).

**TABLE 2:** Results of reliability and convergent validity analyses.

Latent variables		Standard loading	Cronbach’s α	CR	AVE
Perceptions	Item1	0.88	0.95	0.95	0.88
	Item2	0.99			
	Item3	0.92			
Self-efficacy	Item1	0.94	0.93	0.93	0.82
	Item2	0.84			
	Item3	0.93			
LGO	Item1	0.94	0.96	0.96	0.89
	Item2	0.95			
	Item3	0.94			
PGO	Item1	0.87	0.94	0.94	0.83
	Item2	0.95			
	Item3	0.91			
Learning value	Item1	0.92	0.94	0.94	0.84
	Item2	0.91			
	Item3	0.92			

**TABLE 3:** Results of discriminant validity analysis.

Latent variables	Perceptions	Self-efficacy	Learning value	LGO	PGO
Perceptions	0.93				
Self-efficacy	0.21	0.90			
Learning value	0.39	0.07	0.92		
LGO	0.16	0.61	0.07	0.94	
PGO	0.33	0.01	0.76	0.18	0.91

### Structural model

**FIGURE 3:**
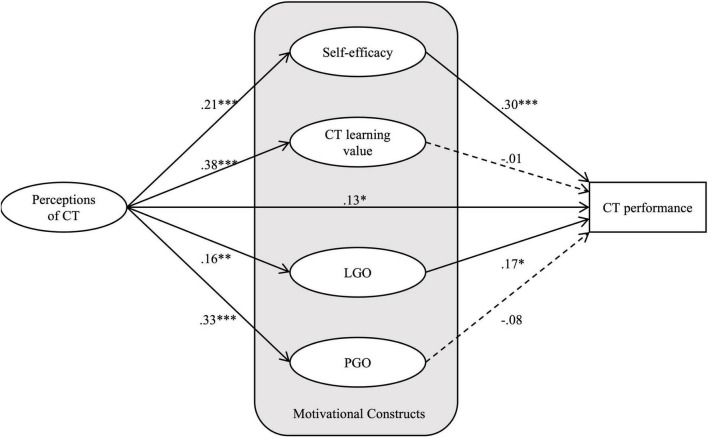
Structural model and standardized path coefficients. *p < 0.05, ^**^p < 0.01, ^***^p < 0.001; dashed lines indicate non-significant paths.

Although the Chi-square result was significant, the SEM results (Figure 3) indicated a good fit of the hypothesized model (χ^2^ = 149.61, *df* = 90, *p* < 0.001, χ^2^/*df* = 1.662, GFI = 0.938, AGFI = 0.906, CFI = 0.987, RMSEA = 0.048). The structural model showed that the students’ perceptions of CT directly and positively influenced their CT performance (β = 0.13, *p* < 0.05). The students’ perception of CT also positively influenced all four motivational constructs: self-efficacy (β = 0.21, *p* < 0.01), PGO (β = 0.33, *p* < 0.01), LGO (β = 0.16, *p* < 0.05), and CT learning value (β = 0.38, *p* < 0.01). As for the relationships between the motivational constructs and the students’ CT performance, the path coefficients showed that the students’ CT performance was positively influenced by self-efficacy (β = 0.30, *p* < 0.01) and LGO (β = 0.17, *p* < 0.05).

### Measurement invariance

The measurement invariance of the latent variables across gender was tested (Table 4). The configural model demonstrated good fit (χ^2^ = 267.65, *df* = 160, *p* < 0.001, GFI = 0.890, AGFI = 0.836, CFI = 0.977, RMSEA = 0.049), suggesting that configural invariance was attained. The model fit indices of the metric and scalar invariance model also showed adequate model fit. For the metric invariance, the increase in χ^2^ was not significant (Δχ^2^ = 13.95, Δ*df* = 10, *p* = 0.175) and the changes in the model indices (ΔCFI = −0.001, ΔTLI = −0.001, ΔRMSEA = −0.001) were within the recommended criteria (Cheung and Rensvold, 2002; Chen, 2007), supporting the metric (weak) invariance. For the scalar invariance, although the increase in χ^2^ was significant (Δχ^2^ = 70.15, Δ*df* = 15, *p* < 0.001), the changes in the model indices (ΔCFI = −0.01, ΔTLI = −0.01, ΔRMSEA = 0.008) were within the recommended criteria, thus supporting the scalar (strong) invariance of the measurement model. We further tested the residual invariance. The increase in χ^2^ was significant, and the changes in the model indices were beyond the recommended criteria, thus rejecting the residual invariance.

**TABLE 4:** Measurement invariance across genders.

	χ^2^	*df*	χ^2^/*df*	CFI	TLI	RMSEA
Configural invariance	267.65	160	1.67	0.977	0.969	0.049
Metric invariance	281.60	170	1.66	0.976	0.970	0.048
Scalar invariance	351.75	185	1.90	0.966	0.960	0.056

### Multigroup comparison

**FIGURE 4:**
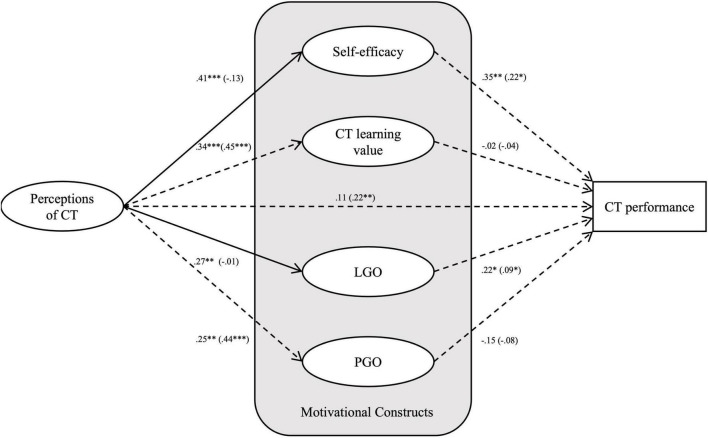
Results of the partially constrained models. *p < 0.05, ^**^p < 0.01, ^***^p < 0.001; β for the female group is in parentheses; solid lines indicate that the structural coefficients differ significantly across gender groups.

The structural invariance test was conducted as follows. First, the model in which factor loading values were constrained and other parameters were freely estimated across gender groups was estimated as the baseline model; it demonstrated good fit (χ^2^ = 313.75, *df* = 195, *p* < 0.001, GFI = 0.880, AGFI = 0.833, CFI = 0.974, RMSEA = 0.046). Second, we tested a fully constrained model in which all of the structural path coefficients were constrained. The comparison results between the fully constrained model and the baseline model (Δχ^2^ = 24.43, Δ*df* = 4, *p* < 0.001) indicated significant differences in certain structural path coefficients across gender groups. Third, the critical ratio test was performed to locate the origin of the structural invariance, and the pairwise parameter values were calculated. A critical ratio statistic greater than +1.96 or smaller than −1.96 indicates differences across genders at the 0.05 significance level. Two path coefficients (perceptions of CT 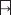 self-efficacy and perceptions of CT 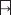 LGO) differed significantly between the male and female students (see Figure 4). The male students who perceived CT more positively showed higher levels of self-efficacy (β = 0.41, *p* < 0.001) and LGO (β = 0.27, *p* < 0.001), whereas the female students’ perception of CT did not have a significant influence on their level of self-efficacy (β = −0.13, *p* = 0.136) or LGO (β = −0.01, *p* = 0.938).

## Discussion

### Perceptions of computational thinking

In this study, students’ perceptions of CT covered three aspects (i.e., perceived impact, perceived interest, and perceived utility). Students who perceive higher levels of these aspects were found to perceive CT as having a greater impact on society, as being more interesting and attractive, and as being more useful to their academic and career development. The students’ perceptions of CT directly influenced their performance on the CT test. This finding is consistent with the findings of Miyazoe and Anderson (2010) and Fabian et al. (2018).

The results also suggest that students with more positive perceptions of CT tend to report higher levels of motivation for CT acquisition. Research has revealed a significant correlation between one’s perception and motivation in different learning contexts (Chen and Hoshower, 2003; Martens et al., 2007). This study contributes to the field by validating this relationship and by further examining the concept of motivation from a multidimensional perspective: students’ perceptions of CT positively influence their self-efficacy, learning value, and goal orientation.

With the implementation of CT in K-12 curricula worldwide, efforts have been made to develop instructional techniques and strategies. This study’s empirical results highlight the need for more research attention on students’ perceptions of CT to further enrich their affective and cognitive learning outcomes, improve the effectiveness of CT acquisition, and facilitate the development of CT education. By examining students’ perceptions from various dimensions, the results yield insights into the development of CT instructional strategies. Furthermore, they imply that more instructional efforts can be made to improve students’ awareness of how CT affects and changes the way people live in modern society, organize attractive and interesting CT learning activities, and engage students in meaningful activities that are relevant to them.

### Motivation and learning performance

In this work, self-efficacy was found to exert a significant effect on CT learning performance, echoing the results of Jaipal-Jamani and Angeli (2017) and Durak (2018). Self-efficacy, as the foundation of human motivation (Bandura, 1986), has been recognized as an effective tool for predicting students’ learning performance in the field of CT. This relationship can be explained by the fact that students who experience CT education through programming constantly encounter problems related to their daily life in programming learning, which leads to greater efforts in their academic study, particularly in their CT course.

In terms of goal orientation, we examined the construct using a two-dimension classification: LGO and PGO. LGO positively influenced the students’ CT performance, whereas PGO did not. The positive relationship between LGO and learning performance is consistent with many previous studies (Gerhardt and Brown, 2006; Hsieh et al., 2007). Researchers have explained that students with high levels of LGO demonstrate more positive self-evaluation and are highly committed to self-improvement and growth, which are all supportive of long-term educational aspirations and “boost” academic achievement. However, research on PGO has generated mixed results. Some researchers have argued that LGO and PGO may go “hand in hand” and that students with high levels of PGO can also have high academic achievements, as they have also reported high levels of commitment and effort (Yukselturk and Bulut, 2005; Polso et al., 2020). Other studies have indicated a negative relationship between PGO and performance (Gerhardt and Brown, 2006). Students with high levels of PGO may experience high levels of stress or depressive symptoms, resulting in lower academic achievements (Tuominen-Soini et al., 2008). Our results show no significant relationship between PGO and performance, which reflects a mixed situation, to some extent, and high levels of commitment and stress may coexist in a group of students with high levels of PGO, which obscures the correlation with CT performance. To examine the effect of PGO more accurately, some researchers have recommended further classifying PGO into performance-approach goal orientation and performance-avoidance goal orientation (Elliot and Harackiewicz, 1996; Schmidt and Ford, 2003). Adopting performance-approach goals means that students aim to demonstrate competence among their peers, and adopting performance-avoidance goals means that they aim to avoid judgments of incompetence. This approach may yield more accurate results for the effects of PGO on learning performance and thus is a potential research direction for CT education development.

The other motivational construct examined in the study is learning value. No significant influence of learning value on CT learning performance was found. This result aligns with some empirical studies (Watson et al., 2014) but contradicts others (Bergin et al., 2005; Román-González et al., 2018). It is generally assumed that students who regard CT as more essential and valuable for their success put more effort into and try to overcome obstacles during their CT learning process. The non-significant association between learning value and CT performance may be attributed to the characteristics of the participating students. The teacher in this high school has constantly emphasized the value of CS in the classroom, and most high school students in the twenty-first century have placed a high value on developing CT skills as well as other learning activities.

### Analysis of multigroup comparison

The measurement invariance results indicate that the measurement model had the same meaning across gender groups and ensured meaningful comparison. The results of the multigroup comparisons reveal that perceptions of CT had a significant positive influence on self-efficacy for the male students but had no significant influence on self-efficacy for the female students. Furthermore, perceptions of CT had a significant positive influence on LGO for the male students but had no significant influence on LGO for the female students. The results suggest that male students tend to exhibit more confidence and more intrinsic impetus in improving their CT skills when they hold positive perceptions of it, whereas female students’ confidence and commitment to self-improvement do not vary significantly along with their perceptions of the learning contents. This finding echoed previous empirical evidence in section “Gender difference” that through interventions of CT, male students exhibited a significant increase in self-efficacy and perceived interest in CT skills (Sullivan et al., 2015; Wei et al., 2021). Previous research has indicated the significant influence of perception of programming on students’ intrinsic motivation (Zainal et al., 2012). We argue that the positive influence of perceptions on self-efficacy and LGO is particularly more salient for male students. This finding provides implications for differentiated instruction such that teachers may devise CT learning activities that promote positive perceptions of the learning content to improve male students’ motivation, whereas there is no special need to consider perceptions in instruction for female students.

## Conclusion

A multigroup SEM analysis was conducted to identify how high school students’ perceptions affect their motivation for CT acquisition and their CT learning performance. Perceptions of CT were found to have a significant effect on CT performance for both male and female students. The students’ perceptions of CT also positively influenced various motivational constructs, including self-efficacy, LGO, PGO, and learning value in CT contexts. Furthermore, the students’ self-efficacy and LGO influenced their CT learning performance, whereas PGO and learning value showed no significant influence on CT learning performance. The multigroup comparison revealed the difference in the correlations between perceptions and motivation across genders. The male students’ self-efficacy and LGO levels were significantly influenced by their perceptions of CT, whereas this correlation was obscured in the female student group.

Our results revealed the important roles of perceptions and motivation in CT education. To cultivate CT talents and improve CT learning effectiveness, CT teaching practices should emphasize the inclusion of students’ affective factors. Increased research attention should also be paid to more diverse tools for measuring perceptions of CT and other related constructs.

Several study limitations must be acknowledged. First, the student participants in this study were recruited from one high school in an urban city in China and might not represent student populations with different backgrounds, thereby limiting the generalizability of the findings to all secondary school students. Future research could include student populations with diverse backgrounds to better predict the relationship. Second, we only included four motivational factors in the measurement; other motivational factors may also play a role in the structural model. To further understand how perceptions and motivation influence each other and also CT learning performance, future studies could include more participants with diverse ethnic and economic backgrounds and incorporate more variables in the measurement model for a more comprehensive understanding. Researchers could also consider using longitudinal data to track changes in students’ CT-related perceptions, motivation, and performance and thus yield more insights into the process of CT acquisition.

## Data availability statement

The original contributions presented in this study are included in the article/supplementary material, further inquiries can be directed to the corresponding author.

## Ethics statement

The studies involving human participants were reviewed and approved by the University of Hong Kong. Written informed consent to participate in this study was provided by the participants’ legal guardian/next of kin.

## Author contributions

JY was responsible for the conceptualization of the study, data collection, formal data analysis, and writing of the manuscript. XL contributed to data analysis and manuscript review and editing. GW contributed to the conceptualization of the study and manuscript review and editing. All authors contributed to the article and approved the submitted version.
